# Polyketides with IDH1 R132h and PTP1B inhibitory activities from the desert-plant-derived fungus *Alternaria* sp. HM 134

**DOI:** 10.3389/fmicb.2022.975579

**Published:** 2022-09-29

**Authors:** Zhuang Li, Lu Meng, Qingyun Ma, Zhen Wang, Youxing Zhao, Duqiang Luo

**Affiliations:** ^1^Key Laboratory of Medicinal Chemistry and Molecular Diagnosis of Ministry of Education, College of Life Science, Hebei University, Baoding, China; ^2^Hainan Key Laboratory of Research and Development of Natural Product from Li Folk Medicine, Hainan Institute for Tropical Agricultural Resources, Institute of Tropical Bioscience and Biotechnology, CATAS, Haikou, China; ^3^Affiliated Hospital of Medical College, Hebei University of Engineering, Handan, China

**Keywords:** desert-plant-derived fungus, polyketides, protein tyrosine phosphatase inhibitory activities, isocitrate dehydrogenase inhibitory activities, *Alternaria* sp.

## Abstract

Five new polyketides named alternafurones A (**1**) and B (**2**), alternapyrones M-O (**3**–**5**), together with fourteen known ones (**6**–**19**), were isolated from the desert-plant-derived fungus *Alternaria* sp. HM 134. The structures of the new compounds were elucidated from spectroscopic data and ECD spectroscopic analyses. Alternafurones A and B represent polyketides with an unprecedented 6/5/6 skeleton core. Compounds **1**, **2** and **4** showed definite inhibitory activities against isocitrate dehydrogenase 1 gene (IDH1 R132h) with IC_50_ values of 29.38, 19.41 and 14.14 μg/ml, respectively. Seven compounds (**6**, **7**, **9**–**12**, **14**) showed potent protein tyrosine phosphatase 1B (PTP1B) inhibitory activity with IC_50_ values ranging from 0.97 μg/ml to 89.80 μg/ml.

## Introduction

Desert regions are extreme ecological environments with large temperature, fluctuations and low precipitation (Eida et al., 2019). The desert has unique extremophiles that aren’t present in other environments and as a result microbes that coevolve in this unique niche may have developed unique or novel secondary metabolites to interact with each other (Yang C. L. et al., 2019; Ameen et al., 2021). Song et al. (2019) obtained the new skeleton dimers from the desert plant endophytic fungus *Trematosphaeria terricola*, which showed antitumor activity. The antibacterial new ansamycin-type polyketides from *Streptomyces* sp. (Rateb et al., 2011) and cytotoxic resorcylic acid lactones (Zhang et al., 2021) from *Chaetosphaeronema hispidulur* were obtained from endophytic fungi of desert environment.

Although some progress had been made in the study of desert fungi, it is still an underdeveloped domain. Thus, we attempted to mine the novel natural products focus on the endophytic fungi in the desert area. An endophytic fungus, *Alternaria* sp. HM 134 was isolated from the endophytic fungi of *Aster pekinensis* Kitag. in Ordos. The *Alternaria* sp. was a recognized plant pathogen, which led to serious crop corruption and a wide range of plant diseases (Zhao et al., 2020). The mycotoxins in *Alternaria* sp. had been widely studied, leading to the definition of a series of secondary metabolites, including polyketones (Lu et al., 2021), dibenzopyrones (Chen et al., 2022), anthraquinones (Chen et al., 2014) and cephalochromin (Song et al., 2021). These metabolites have cytotoxic, antiviral and enzyme inhibitory activities (Xu et al., 2019; Tan et al., 2020; Saxena, 2021; Zhang et al., 2022).

In this study, chemical investigation on the fermentation broth of *Alternaria* sp. HM 134 led to the identification of five new polyketides (**1**–**5**) and fourteen known ones (**6**–**19**). All the isolated compounds were tested for their inhibitory activities against isocitrate dehydrogenase (IDH) and potent protein tyrosine phosphatase (PTPs). Compound **9** has significant inhibitory activity against PTP1B. Herein, the details of the isolation, structure identification, and bioactivity of these compounds were described.

## Materials and methods

### General experimental procedure

Ultraviolet (UV) spectra were measured on a UV-3600 spectrometer. ECD spectra were measured on Bio-Logic MOS-450 spectropolarimeter. IR spectra were recorded on a Thermo Nicolet iS 10 spectrometer. The NMR spectra were recorded on a Bruker AM-600 spectrometer with TMS as an internal standard. HRESIMS spectra were obtained on a Thermo U3000 spectrometer fitted with an ESI source. Semipreparative HPLC was performed on Thermo UltiMate 3,000 machine equipped with a 5C18-MS column (5 μM, 250 × 10 mm,COSMOSIL, JPN). ECD spectroscopic analyses was supported by the High-Performance Computing Center of Hebei University.

### Fungal material

The HM 134 was isolated from the desert plant *Aster pekinensis* Kitag. in Ordos. The strain was identified as *Alternaria* sp. based on microscopic examination and by internal transcribed spacer (ITS1-4) sequencing. The ITS sequence has been deposited in GenBank^1^ with accession number No. MK478900. The purified strain was cultivated in a PDA medium plate (containing 200 g potatoes, boil 20 min, take filtrate; 20 g glucose; 20 g agar in 1 l water) at 28°C for 7 days. Then, it was cut into small pieces and cultured in PDB medium plate (containing 200 g potatoes, boil 20 min, take filtrate; 20 g glucose;in 1 l water) for 5 days.

### Fermentation, extraction, and isolation

The fermentation was carried out in 100 flasks (500 ml), each containing 80 g of rice and 100 ml H_2_O, autoclaving at 15 psi for 30 min. After cooling to room temperature, each flask was inoculated with 20 ml of the spore inoculum and incubated at room temperature for 30 days. The fermented material was extracted successively with EtOAc for three times, then the EtOAc solutions were combined and evaporated under reduced pressure to get 170 g of crude extract. The extract was fractionated by a silica gel VLC column using different solvents of increasing polarity, from EtOAc-petroleum to yield eight fractions (Fr.1–8). Fraction Fr.3 was chromatographed over C18 reversed-phase (RP-18) silica gel using MeOH/H_2_O (30:70, 40:60, 50:50, 60:40, 70:30, 90:10) to collect six subfractions (SF.3a − 3f). SF.3d was separated on a semipreparative reversed-phase (RP) HPLC column using MeOH/H_2_O = 60:40 (2.5 ml/min) to give **4** (*t*_R_ = 16.3 min, 3 mg). SF.3c was separated on a semipreparative reversed-phase (RP) HPLC column using MeOH/H_2_O = 40:60 (2.5 ml/min) to give **5** (*t*_R_ = 18.6 min, 2 mg). The precipitated compound **3** was obtained in SF.3e. Fraction Fr.4 was chromatographed over C18 reversed-phase (RP-18) silica gel using MeCN/H_2_O (10,90, 20:80, 30:70, 40:60, 50:50, 60:40, 70:30) to collect seven subfractions (SF.4a − 4 g). SF.4b was separated on a semipreparative reversed-phase (RP) HPLC column using MeCN/H_2_O = 18:82 (2.5 ml/min) to give **1** (*t*_R_ = 21.2 min, 3 mg). SF.4b was separated on a semipreparative reversed-phase (RP) HPLC column using MeCN/H_2_O = 18:82 (2.5 ml/min) to give **2** (*t*_R_ = 22 min, 2 mg).

### Spectroscopic data

*Alternafurone A (**1**):* Yellow oil; [*α*]^25^_D_ + 57.3 (*c* 0.1, MeOH); UV (MeOH) *λ*_max_ (log *ε*): 218 (5.18), 257 (2.01), 293 (0.78) nm; IR (KBr) *v*_max_ cm^−1^: 3422, 2,945, 1,698, 1,672, 1,512, 1,458, 1,016. ^1^H and ^13^C NMR data see Table 1; HRESIMS *m/z* 291.0876 [M - H]^−^ (calcd for C_15_H_15_O_6_^−^, 291.0874).

**Table 1:** ^1^H (600 MHz) and ^13^C NMR (150 MHz) data of compounds (1–2).

No.	1		2	
	*δ*_H_ (*J* in Hz)	*δ*_C_, type	*δ*_H_ (*J* in Hz)	*δ*_C_, type
1		171.0, C		169.1, C
2		105.9, C		106.4, C
3		159.7, C		159.7, C
4	6.44, s	102.7, CH	6.42, s	103.0, CH
5		168.8, C		168.9, C
6	6.39, s	100.1, CH	6.41, s	99.8, CH
7		157.8, C		157.6, C
8		87.3, C		88.2, C
9	1.98, d (12.4) 2.47, m	38.8, CH_2_	2.01, dd (14.0, 3.6) 2.16, t (13.1)	43.4, CH_2_
10	4.13, d (8.8)	67.1, CH	3.87, m	71.2, CH
11	4.25, s	67.3, CH	4.07, dd (8.0, 2.2)	74.0, CH
12	5.86, d (3.3)	130.1, CH	5.74, s	134.4, CH
13		136.9, C		132.9, C
14	1.4, s	16.6, CH_3_	1.31, s	16.9, CH_3_
5-OCH_3_	3.84, s	56.6, OCH_3_	3.85, s	56.6, OCH_3_

*Alternafurone B (**2**):* Yellow oil; [*α*]^25^_D_ + 49.4 (*c* 0.1, MeOH); UV (MeOH) *λ*_max_ (log *ε*): 246 (4.20), 278 (1.62), 320 (0.99) nm; IR (KBr) *v*_max_ cm^−1^: 3455, 2,963, 1714, 1,675, 1,516, 1,445, 1,033. ^1^H and ^13^C NMR data see Table 1; HRESIMS *m/z* 291.0879 [M - H]^−^ (calcd for C_15_H_15_O_6_^−^, 291.0874).

*Alternapyrone M (**3**):* White solid; [*α*]^25^_D_ + 39.1 (*c* 0.1, MeOH); UV (DMSO) *λ*_max_ (log *ε*): 243 (2.86), 278 (0.88), 316 (0.49) nm; IR (KBr) *v*_max_ cm^−1^: 3526, 1710, 1,663, 1,618, 1,463, 1,012. ^1^H and ^13^C NMR data see Table 2; HRESIMS *m/z* 361.0932[M - H]^−^ (calcd for C_18_H_17_O_8_^−^, 361.0929).

**Table 2:** ^1^H (600 MHz) and ^13^C NMR (150 MHz) data of compounds (3–5).

No.	3 (in DMSO-*d*_6_)		4 (in CD_3_OD-*d*_4_)		5 (in CD_3_OD-*d*_4_)	
	*δ*_H_ (*J* in Hz)	*δ*_C_, type	*δ*_H_ (*J* in Hz)	*δ*_C_, type	*δ*_H_ (*J* in Hz)	*δ*_C_, type
1		167.9, C		169.7, C		170.6, C
2		100.4, C		101.6, C		101.6, C
3		162.9, C		165.4, C		165.6, C
4	6.55, d, (2.3)	101.7, CH	6.5, s	102.3, CH	6.21, s	101.8, CH
5		165.9, C		167.9, C		166.6, C
6	6.80, d, (2.2)	102.8, CH	6.68, s	104.0, CH	6.27, s	105.1, CH
7		138.2, C		139.1, C		145.0, C
8		136.7, C		134.0, C	3.14, d, (9.6)	43.4, CH
9	6.37, s	129.2, CH	6.27, s	129.0, CH	1.70, q, (12.3) 2.22, dt, (13.7, 3.9)	28.5, CH_2_
10		79.8, C		106.3, C	3.86, dt, (12.0, 4.0)	72.2, CH
11	3.90, m	68.0, CH		77.2, C	4.11, d, (3.5)	70.0, CH
12	2.08, overlap 2.25, dd, (14.6, 4.2)	38.6, CH_2_	2.52, m	47.0, CH_2_	2.05, dd, (14.0, 2.3) 2.24, dd, (14.0 3.0)	43.5, CH_2_
13		80.6, C		83.9, C		84.7, C
14	1.47, s	27.7, CH_3_	1.59, s,	27.0, CH_3_	1.36, s	20.9, CH_3_
15		177.1, C	4.85, overlap	75.6, CH		
16	4.58, td, (9.3, 6.3)	66.9, CH	2.32, dd, (13.2, 10.2) 2.57, dd, (13.2, 6.5)	44.3, CH_2_		
17	2.12, overlap 2.72, dd, (13.0, 8.9)	41.0, CH_2_		174.3, C		
						
5-OCH_3_	3.88, s	56.0, CH_3_	3.88, s	56.4, CH_3_		
17-OCH_3_			3.70, s	52.7, CH_3_		
3-OH	11.14, s					
10-OH						
11-OH	5.63, d, (5.2)					
16-OH	6.04, d, (6.1)					

*Alternapyrone N (**4**):* Brown oil; [*α*]^25^_D_ -13.2 (*c* 0.1, MeOH); UV (MeOH) *λ*_max_ (log *ε*): 208 (2.76), 271 (2.05), 295 (1.05) nm; IR (KBr) *v*_max_ cm^−1^: 3428, 2,948, 1717, 1,578, 1,438, 1,162. ^1^H and ^13^C NMR data see Table 2; HRESIMS *m/z* 391.1039[M - H]^−^ (calcd for C_19_H_19_O_9_^−^, 391.1035).

*Alternapyrones O (**5**):* Pink oil; [*α*]^25^_D_ -9.1 (*c* 0.1, MeOH); UV (MeOH) *λ*_max_ (log *ε*): 213 (2.80), 269 (1.13), 302 (0.62) nm; IR (KBr) *v*_max_ cm^−1^: 3679, 2,942, 1718, 1,586, 1,435, 1,014. ^1^H and ^13^C NMR data see Table 2; HRESIMS *m/z* 279.0873[M - H]^−^ (calcd for C_14_H_15_O_6_^−^, 279.0876).

### Computational section

The initial configuration was retrieved by Molecular Operating Environment (MOE) using MMFF94 molecular mechanics force field. Gauss 16 software was used for density functional theory calculation (Frisch et al., 2019). These conformations were optimized with B3LYP/6-31G (d) in gas phase, and the conformations with Boltzmann-population of over 1% were retained for the next operation. The remaining configurations were further optimized with B3LYP/6-31G (d) in gas phase, and all configurations were guaranteed to be frequency analyzed at the same level to avoid repeated configurations. The ECD spectra were calculated by the Time-dependent Density functional theory (TDDFT) methodology at the B3LYP/6–31 + g (d, p) level in methanol. ECD spectra were simulated using SpecDis 1.71 (Bruhn et al., 2013) with *σ* = 0.30 eV.

### Inhibition of protein tyrosine phosphatase (PTPs) assay

Protein tyrosine phosphatase (PTPs) plays an important role in many human threatening diseases, such as PTP1B, an effective target for the treatment of type II diabetes; CD45, an effective target for the treatment of leukemia; and TCPTP, a reliable target for the treatment of cancer involved in multiple signaling pathways (Ruddraraju and Zhang, 2017). Nitrophenyl phosphate (pNPP) was used as substrate to determine the enzyme inhibition test. Fifty microliter reaction buffer (pH 6.5) including 50 mM HEPES, 100 mM NaCl, 1 mM EDTA, and 1 mM dithiothreitol (DTT) and compounds was added to 96 well plates, and incubate at room temperature for 15 min. Further, 50 μl reaction buffer with 50 mM pNPP was added and incubated at 37°C for 60 min. Na_3_VO_4_ was used as positive control and DMSO as the negative control. The phosphatase activity was determined by absorbance measured at 405 nm. The test was repeated three times for each compound. The IC_50_ value was derived from three independent experiments.

### Isocitrate dehydrogenase inhibition assay

IDH1 R132h model was used to screen the inhibitory activity of the compounds. DMSO was used to configure the compounds into different concentrations as test samples. 2 mm NADPH (3 μl) and 1 m *α*-Kg (0.1 μl) was added to IDH1 buffer (pH 6.5; 96 μl) solution as reaction substrate in the reaction system. Then, the test samples (2.0 μl), IDH1 R132h (0.4 μl), PMS (0.1 μl) and 15 mM WST-8 (1.0 μl) were successively added to the above mixture and reacted at room temperature for 60 min. AGI-5198 was used as positive control and DMSO as the negative control. The absorption peak of 450 nm was detected by microplate reader. The test was repeated three times for each test samples, and the IC_50_ was calculated by GraphPad Prism.

## Results and discussion

### Isolation and structure elucidation

**Figure 1:**
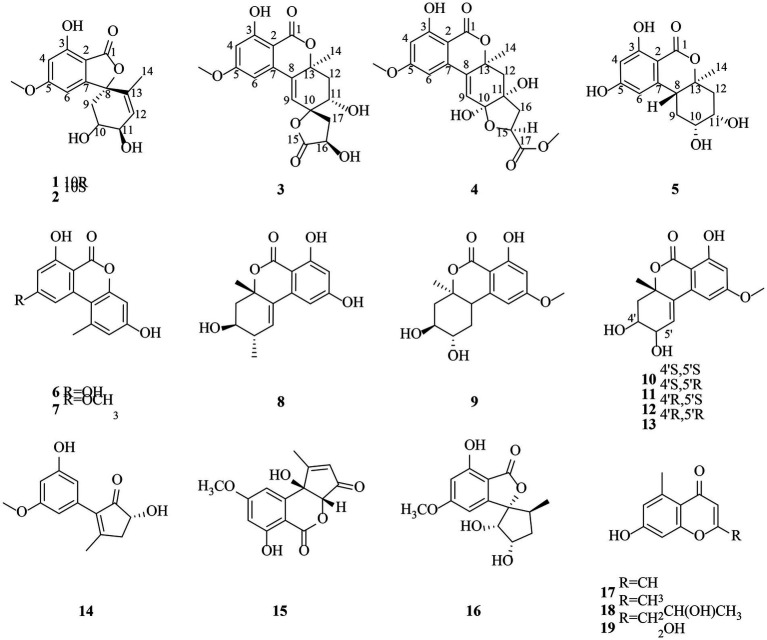
Structures of compounds **1–19**.

The fungus *Alternaria* sp.HM 134 was isolated from the desert plant *Aster pekinensis* Kitag. in Ordos and identified by internal transcribed spacer (ITS1-4) sequencing. The strain was grown in solid medium containing 80 g of rice and 100 ml H_2_O and incubated at room temperature for 30 days. The fermented material was extracted with EtOAc and the crude extract was separated and purified by a variety of separation and analysis methods. Finally, 19 natural products were isolated and identified from this fungus (Figure 1), including five new polyketides named alternafurones A (**1**) and B (**2**), alternapyrones M-O (**3**–**5**), and were tested for their inhibitory activities against PTPs and IDH for the first time.

**Figure 2:**
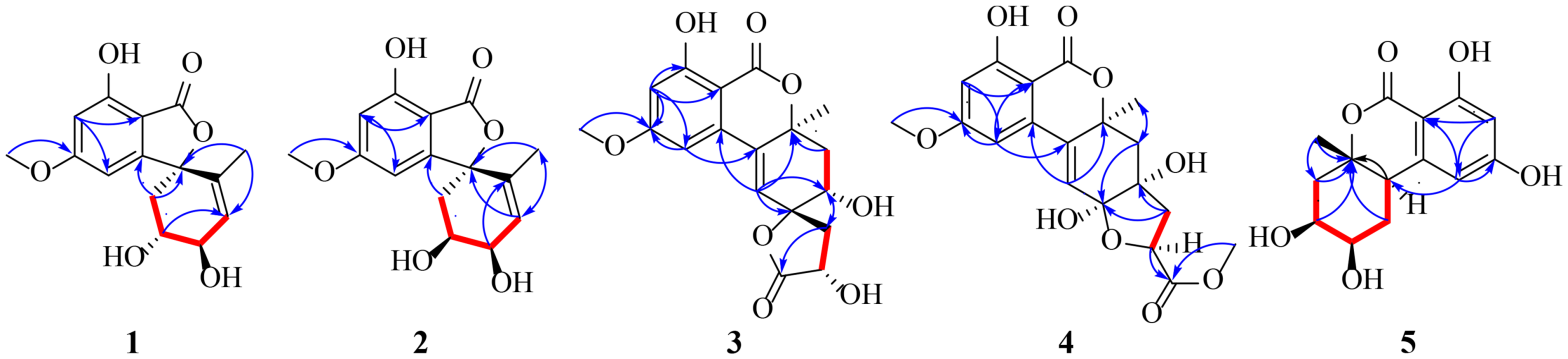
Key COSY (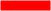) and HMBC (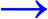) correlations of **1-5**.

**Figure 3:**
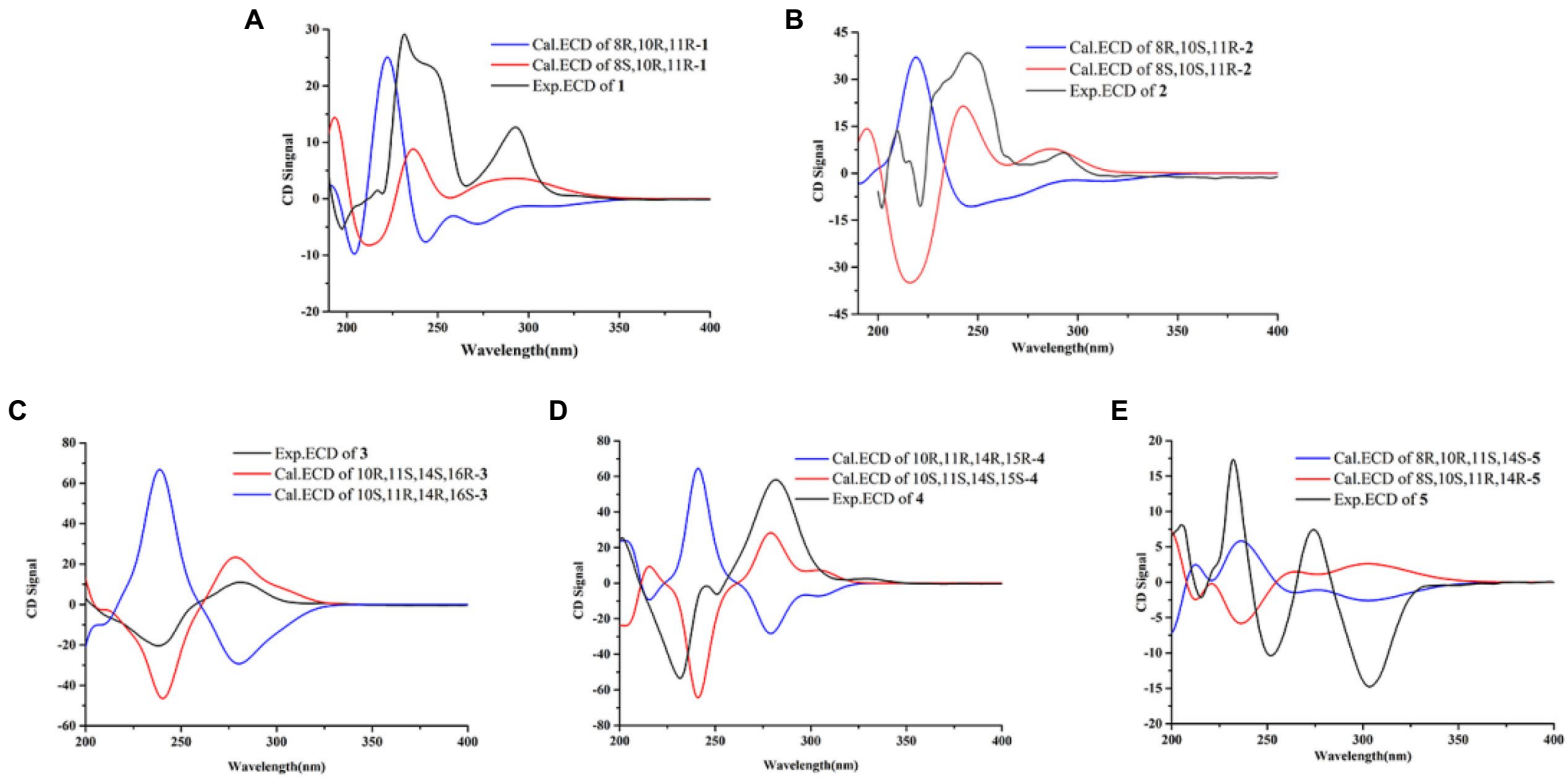
Experimental and calculated ECD curves for compound 1 **(A)**, compound 2 **(B)**, compound 3 **(C)**, compound 4 **(D)**, compound 5 **(E)**.

Compound **1** was obtained as yellow oil, and its molecular formula was determined to be C_15_H_16_O_6_ by HRESIMS and NMR data (Table 1), indicating an index of hydrogen defificiency of 8. The ^1^H NMR spectrum indicated two aromatic protons [*δ*_H_ 6.44 (H-4) and 6.39 (H-6)], an olefinic proton (*δ*_H_ 6.44,H-12), one methoxy (*δ*_H_ 3.84, H-15), and one methyl (*δ*_H_ 1.40, H-14). The ^13^C NMR spectrum revealed 15 carbon resonances that were classified by HSQC spectrum as, one carbonyl, one methyl, one methoxy, one methylene, five methines (two oxygenated and three orefinic) and six nonprotonated carbons. A comparison of the NMR data of **1** with those of alternatains A (**16**; Pang et al., 2018) indicated that they shared similar isobenzofuranone skeleton, except that the five-membered ring *via* the spiro carbon C-8 in alternatains A was replaced by a six-membered ring. This deduction was confirmed by HMBC correlations (Figure 2) from H-9 (*δ*_H_ 1.98, 2.47) to C-7 (*δ*_C_ 157.8) and C-8 (*δ*_C_ 87.3), from H-12 (*δ*_H_ 5.86) and H-14 (*δ*_H_1.40) to C-8, as well as sequential COSY correlations of H-9/H-10/H-11/H-12. The whole connectivity of 6/5/6 skeleton core in **1** was further demonstrated by other HMBC correlations (Figure 2) and analysis of its molecular formula. The linkage of methoxy (*δ*_H/C_ 3.84/56.6) at C-5 was based on HMBC correlations from methoxy protons to C-5 and from H-6 (*δ*_H_ 6.39) to C-8 (*δ*_C_ 87.3) and C-5. Thus, the gross structure of **1** was resolved as shown. The relative configuration was established by analysis of ROESY data. According to the ROESY spectrum, the absence of ROESY correlation between H-10 (4.13, 1H) and H-11 (4.25, 1H) suggested that 10-OH is opposite to 11-OH. In order to ascertain the absolute configuration of **1**, its electronic circular dichroism (ECD) spectrum was determined in MeOH and simulated at the CAM-B3LYP/6–311++G(2d, p) level after conformational optimization at the same level *via* Gaussian 05 software. The Boltzmann-weighted ECD curve agreed well with the experimental one (Figure 3A), and the absolute configurations of stereocenters C-8, C-10, and C-11 were assigned to be 8*S*,10*R*,11*R*.

**Figure 4:**
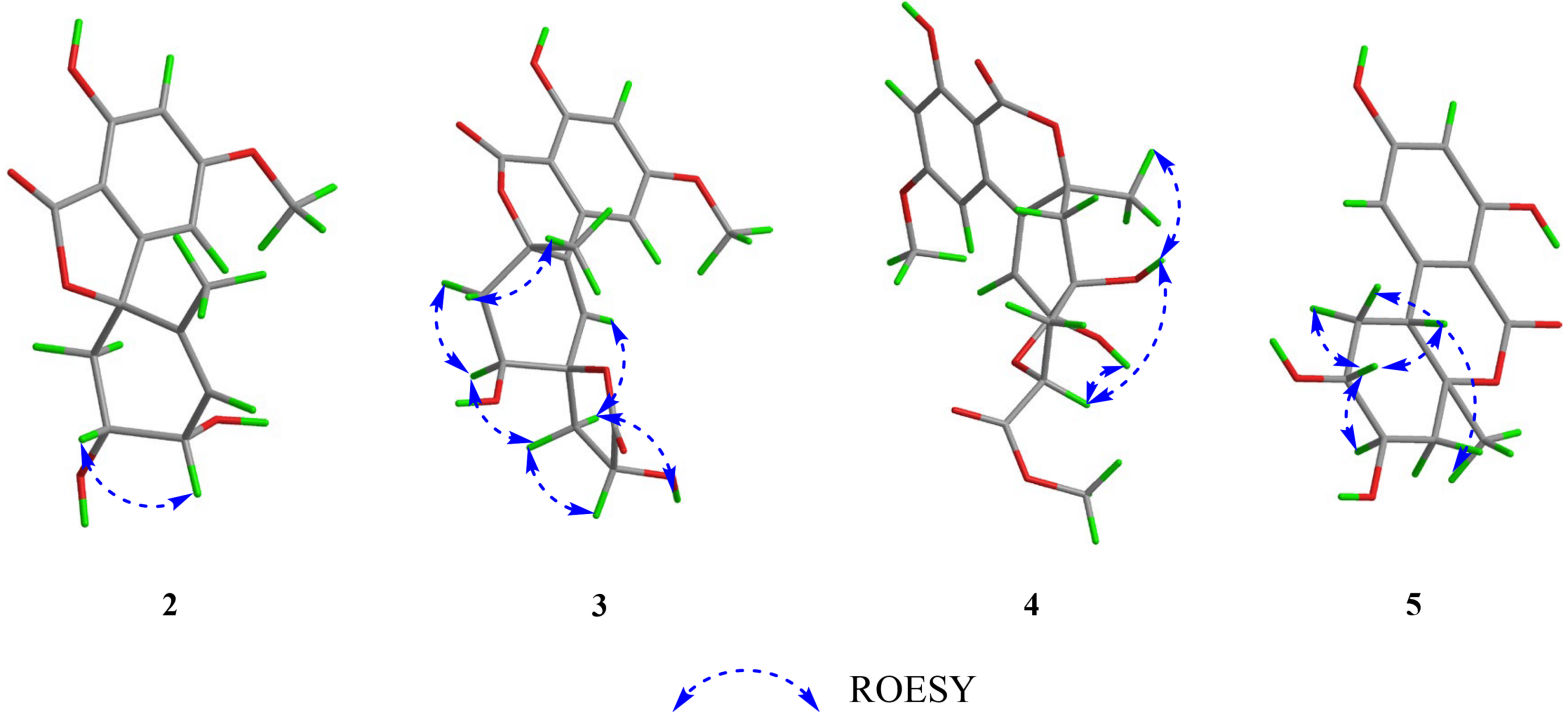
Selected ROESY correlations of compounds **2-5**.

Compound **2** had the same molecular formula C_15_H_16_O_6_ as that of **1** by HRESIMS and NMR data (Table 1) Further comparing its closely similar NMR data with those of compound **1** suggested that they possessed the same planar structure with 6/5/6 skeleton core. The main difference of their NMR data was reflected in the chemical shifts of C-9-C-13 in six-membered ring, which may be resulted from the different configuration of C-10 and C-11. The whole connectivity of compound **2** was also further demonstrated by other HMBC correlations (Figure 2). The relative configuration of six-membered ring was established by analysis of ROESY data. The obvious ROESY correlation of H-10 (3.87, 1H) and H-11 (4.07, 1H) indicated the same face of these two protons (Figure 4). The calculated ECD curve for (8*S*,10*S*,11*R*)-**2** matched well with the experimental spectrum (Figure 3B), assigning the 8*S*,10*S*,11*R* absolute configuration of **2**.

The molecular formula of compound **3** was determined to be C_18_H_18_O_8_ by HRESIMS and NMR data (Table 2), indicating 10 degrees of unsaturation. The ^1^H and ^13^C NMR data of **3**, with the aid of a HSQC spectrum, showed a total of 18 carbon signals comprising two ester carbonyls, eight olefinic or aromatic carbons with three protonated, two sp^3^ methylenes, two sp^3^ oxygenated methines, two sp^3^ non-protonated oxygenated carbons, one methoxy and one methyl. Detail analyses of the 1D and 2D NMR data revealed that the structure of **3** had a 6/6/6 skeleton system, which was similar to compound alternatain D (Yang H. Y. et al., 2019). The obvious structural difference between them is that the carboxyl group in alternatain D was replaced an ester carbonyl *via* OH-10 to firm the five-membered spiro ring in **3**. This deduction was supported by the COSY cross-peaks (Figure 2) of OH-11/H-11/H-12 and H-16/H-17, combined the analysis of its molecular formula. The whole connectivity of compound **3** with 6/6/6/5 skeleton system was also further demonstrated by other HMBC correlations from H-16 (*δ*_H_ 4.58) to C-15 (*δ*_C_ 177.1) and C-17 (*δ*_C_ 41.0), from H-14 to C-8, C-13 and C-12. The relative configuration was established by analysis of ROESY data, revealing the key correlations of H-17β (2.12) with H-9 (6.37) and 16-OH, H-17α (2.72) with H-16 (4.58) and H-11, H-12β (2.25) with H-11 and H-12α (2.08) with H-14 (Figure 4). These ROESY correlations showed the relative configuration of **3** as shown in Figure 4, suggesting that its absolute configuration was determined as 10*R*,11*S*,14*S*,16*R* or 10*S*,11*R*,14*R*,16*S* (Figure 4). Comparison of its experimental ECD spectrum with the calculated data of **3** (Figure 3), the absolute configuration of compound **3** was finally determined as 10*R*,11*S*,14*S*,16*R*.

Compound **4** had the molecular formula C_19_H_20_O_9_ as established from its HRESIMS and NMR data (Table 2), indicative of an index of hydrogen deficiency of 10. Its ^1^H NMR spectrum showed three olefinic or aromatic protons [*δ*_H_ 6.50 (H-4), 6.68 (H-6), 6.27 (H-9)], one methyl (*δ*_H_ 1.59, H-14), two methoxys (*δ*_H_ 3.88 and 3.70). The ^13^C NMR data of **4** showed a total of 19 carbon signals comprising two ester carbonyls, eight olefinic or aromatic carbons with three protonated, two sp^3^ methylenes, two sp^3^ oxygenated methines, two sp^3^ non-protonated oxygenated carbons, two methoxys and one methyl. Detail comparison of its 1D and 2D NMR data with that of alternatain C (Yang H. Y. et al., 2019) showed that they had the same 6/6/6/5 skeleton system. The structural difference between them is that the hemiketal hydroxy at C-15 in alternatain C was linked at C-10 and one additional methyl connected the carboxyl group to firm ester carbonyl in **4**. This deduction was comfirmed by the key HMBC correlations from H-9 and H-12 to C-10, from the methoxy protons to C-17, and the COSY cross-peaks (Figure 2) of H-15/H-16. The whole connectivity of compound **4** was also further demonstrated by other HMBC correlations (Figure 2). The ROESY spectrum of compound **4** was obtained by dissolution of the sample with DMSO (see Supplementary Material, Figure S27), and the key correlations of 10-OH (5.78, 1H)/H-15 (4.72, 1H), H-15/11-OH (6.17, 1H), and 11-OH/H-14 (1.51, 3H) assigned its relative configuration (Figure 4). The absolute configuration of **4** was determined based on comparing its experimental ECD spectrum with those of calculated data (Figure 3D), indicating the stereocenters of 10*S*,11*S*,14*S*,15*S*.

The molecular formula of compound **5** was determined to be C_14_H_16_O_6_ by HRESIMS and NMR data (Table 2), establishing an index of hydrogen deficiency of 7. ^1^H NMR showed two aromatic protons [*δ*_H_ 6.27 (1H, s, H-6), 6.21 (1H, d, H-4)] and one methyl [*δ*_H_ 1.36 (3H, s, 14-CH_3_)]. The ^13^C NMR spectrum revealed 14 carbon resonances that were classified by HSQC spectrum as one carbonyl, one methyl, two sp^3^ methylenes, five methines (two olefinic and two oxygenated) and five nonprotonated carbons. Analyses of the NMR data revealed that the structure of **5** was closely similar to dihydroaltenuene A (Jiao et al., 2006). The only difference between them was that the methoxy at C-8 in dihydroaltenuene A was replaced by a hydroxy in **5**, which was comfirmed by the molecular weight difference 14 and the HMBC correlation of the methoxy with C-8. The relative configuration was established by the key ROESY correlations of H-11 (4.11)/H-10 (3.86), H-10/H-8 (3.14), H-9α/H-8 and H-9β/H-14 (1.36). The absolute configuration was assigned to be 8*R*,10*R*,11*S*,13*S* by comparison of its experimental ECD spectrum with the calculated ECD curves of **5** (Figure 3E).

In addition to the five compounds described above, we also isolated 14 known ones (Figure 1), alternariol (**6**; Tanahashi et al., 1997), alternariol 9-methyl ether (**7**; Meng et al., 2012) altenuene (**8**; Xiao et al., 2014), 3-epidihydroaltenuene A (**9**; Tian et al., 2016), 5-hydroxyepialtenuene (**10**; Jin et al., 2013), 5′-epialtenuene (**11**; Bradburn et al., 1994), 4′-epialtenuene (**12**; Aly et al., 2008) isoaltenuene (**13**; He et al., 2012), (+)-nigrosporaol A (**14**; He et al., 2016), (3a*R*,9b*R*)-6,9b-dihydroxy-8-methoxy-1-methyl-cyclopentene[c]isochromen-3,5-dion (**15**; Jiao et al., 2013), alternatains A (**16**; Yang H. Y. et al., 2019), 2,5-dimethyl-7-hydroxychromone (**17**; Kashiwada et al., 2008), 2-(2’*S*-hydroxypropyl)-5-methyl-7-hydroxychromone (**18**; Khamthong et al., 2012), 7-hydroxy-2-hydroxymethyl-5-methyl-4H-chromen-4-one (**19**; Kimura et al., 2008). All of these compounds were identified by comparing their ^1^H and ^13^C NMR data with those reported in the literatures.

### Protein tyrosine phosphatase inhibition assay

Protein tyrosine phosphatase (PTPs) plays an important role in many human threatening diseases, such as PTP1B, an effective target for the treatment of type II diabetes; CD45, an effective target for the treatment of leukemia; and TCPTP, a reliable target for the treatment of cancer involved in multiple signaling pathways (Zhang, 2003; Vintonyak et al., 2009). All the compounds were tested for their *in vitro* inhibitory activities against PTP1B, CD45 and TCPTP. The results showed that the five new compounds had no inhibitory activity against the three enzymes of the PTPs family compared with Na_3_VO_4,_ as shown in Table 3. Compounds **9** and **12** had inhibitory activity against PTP1B compared with other compounds. It was noteworthy that the IC_50_ value (0.97 μg/ml) of compound **9** was similar to the positive control with IC_50_ of 0.58 μg/ml. Compound **6** and **8** had definite inhibitory activity against TCPTP and CD45. For the three enzymes, compound **6** had a wide range of inhibitory activities.

**Table 3:** Inhibitory activities of compounds against PTPs.

Compound	IC_50_(μg/ml)		
	PTP1B	TCPTP	CD45
**1–5**	>100	>100	>100
**6**	15.75 ± 0.74	7.79 ± 0.32	5.80 ± 0.08
**7**	37.86 ± 2.22	57.38 ± 7.26	19.82 ± 0.49
**8**	>100	10.42 ± 1.50	4.55 ± 0.82
**9**	0.97 ± 0.13	>100	>100
**10**	45.63 ± 3.02	>100	>100
**11**	54.54 ± 7.32	>100	>100
**12**	7.44 ± 0.25	>100	>100
**14**	89.80 ± 2.12	>100	>100
**17**	>100	>100	19.73 ± 0.85
**19**	>100	>100	24.78 ± 1.02
Na_3_VO_4_^a^	0.58 ± 0.06	0.44 ± 0.11	0.36 ± 0.09

a *Positive control for against PTPs*.

### Biological activity against isocitrate dehydrogenase

We have learned that isocitrate dehydrogenase (IDH) gene mutations are closely related to the occurrence and development of tumors (Fujii et al., 2016). IDH mutations mainly occur in malignant tumors such as glioma, acute myeloid leukemia, chondrosarcoma, and intrahepatic cholangiocarcinoma. IDH mutation ultimately promote the occurrence and development of tumors (Gondim et al., 2019). Therefore, inhibition of IDH1 R132h activity was screened using the above compounds. AGI-5198 was used as a positive control in this experiment with the IC_50_ value of 0.04 μg/ml. The results showed that compounds **1**, **2** and **4** had a low level of IDH1 R132h inhibitory activities, with IC_50_ of 29.38, 19.41 and 14.14 μg/ml, as shown in Table 4.

**Table 4:** Inhibitory activities of compounds against IDH1-R132H.

Compound	IC_50_ (μg/ml)
**1**	29.38 ± 0.87
**2**	19.41 ± 0.33
**3**	>53.3
**4**	14.14 ± 0.25
**5–19**	>53.3
AGI-5198^a^	0.04 ± 0.002

a *Positive control for against IDH1-R132H*.

## Conclusion

In this work, nineteen polyketides, including 5 new ones, were isolated from the endophytic fungus *Alternaria* sp. HM134 from the desert plant *Aster pekinensis* Kitag. in Ordos. Although most isolated polyketides have been found in the genus *Alternaria* in non-desert environments, the skeleton of alternafurones A (**1**) and B (**2**) comprised an unprecedented 6/5/6 skeleton core, which enriched the diversity of polyketone chemical structure. Meanwhile, three compounds (**1**, **2** and **4**) showed inhibitory activities against IDH1 R132h. Compounds **6**–**8** had inhibitory activity against TCPTP and five compounds (**6**–**8**, **17**, **19**) showed inhibitory activity against CD45. Seven compounds (**6**, **7**, **9**–**12**, **14**) showed inhibitory activity against PTP1B, among which compound **9** had a specific and significant inhibition. Furthermore, polyketides are reported with significant PTP1B inhibitory activity for the first time, which may provide new options for the development of therapeutic agents for diabetes and cancer.

## Data availability statement

The original contributions presented in the study are included in the article/Supplementary material, further inquiries can be directed to the corresponding authors.

## Author contributions

DL, YZ, and ZW contributed to the conception and design of the study. LY and FK determined the plane structure and absolute configuration. ZL and LM wrote the first draft of the manuscript and performed all of the experimental work. QM contributed to isolation of compounds. ZL contributed to bioactivity assay. DL, YZ, and ZL improved the manuscript. All authors contributed to manuscript revision as well as read and approved the submitted version.

## Funding

This research was supported by Youth program of National Natural Science Foundation (32100569), Financial Fund of the Ministry of Agriculture and Rural Affairs, P. R. of China (NFZX2021) and Post-graduate’s Innovation Fund Project of Hebei University (HBU2021bs005).

## Conflict of interest

The authors declare that the research was conducted in the absence of any commercial or financial relationships that could be construed as a potential conflict of interest.

## Publisher’s note

All claims expressed in this article are solely those of the authors and do not necessarily represent those of their affiliated organizations, or those of the publisher, the editors and the reviewers. Any product that may be evaluated in this article, or claim that may be made by its manufacturer, is not guaranteed or endorsed by the publisher.

## Footnotes

^1^ http://www.ncbi.nlm.nih.gov
